# Evaluation of Large Language Models as Tools, Models, and Partners in Creative Thinking Research: A Selective Narrative Review with the GCA Framework

**DOI:** 10.3390/jintelligence14090218

**Published:** 2026-09-11

**Authors:** Kexin Huang, Chunlei Liu, Jiaqin Yang

**Affiliations:** 1 School of Psychology, Qufu Normal University, Qufu 273165, China; 2 School of Psychology, University of Nottingham, Nottingham NG7 2RD, UK

**Keywords:** large language models, creativity, divergent thinking, automated assessment, human–AI co-creativity, semantic distance, cognitive modelling, intelligence

## Abstract

Creativity research faces three persistent bottlenecks: divergent-thinking scoring is labour-intensive, cognitive models of creativity remain underspecified, and laboratory tasks fall short of real-world creative achievement. Large language models (LLMs) offer potential solutions, but the field lacks a structured framework for evaluating them. This selective narrative review (January 2018–June 2026) applies the generation–capability–assessment (GCA) framework, whose three axes are operationalised through descriptive criteria with provisional heuristic thresholds. On the generation axis, LLMs exceed average human performance on divergent-thinking tasks in most independent comparisons (Hedges’ g ≈ 0.5–2.6), an advantage qualified by fluency dependency, the superiority of top-performing humans at scale, and a novelty–typicality trade-off. On the capability axis, LLMs simulate some task-level associative behaviour and can generate hypotheses for human research, but there is no evidence that they instantiate human-like creative mechanisms. On the assessment axis, automated scoring shows promising reliability and convergent validity for specific languages and tasks (ICC ≥ 0.80 and r ≥ 0.70 in selected studies), but cross-language generalisation is largely untested and individual-level use is unsupported. Human–AI co-creativity may benefit from a division of labour, although social–affective dimensions may matter more than cognitive support. We provide a GCA reporting protocol and identify research priorities.

## 1. Introduction

Creative thinking—the capacity to generate ideas that are both novel and useful (Runco & Jaeger, 2012)—has been studied in cognitive psychology for decades. The field has produced rich theoretical frameworks, including the dual-process model of divergent and convergent thinking (Guilford, 1950; Cropley, 2006), Mednick’s (1962) associative theory, and the Geneplore model of generative and exploratory processes (Finke et al., 1992), and neurocognitive research has mapped the default-mode and executive-control networks that support creative thought (Beaty et al., 2018). Yet three bottlenecks persist: manual scoring of divergent-thinking tasks is labour-intensive and requires trained raters (Amabile, 1982); cognitive models of creative processes remain underspecified at the computational level; and laboratory creativity tasks remain far removed from real-world creative achievement (Kaufman & Beghetto, 2009).

Large language models (LLMs) such as GPT-4, Claude, and LLaMA offer a potential resolution: they can generate hundreds of creative responses in seconds, compute semantic-distance scores that correlate with human judgements of originality, and serve as tractable models of associative processes (Beaty et al., 2022; Beaty & Kenett, 2023). But the literature is fragmented and often contradictory: some studies report LLMs outperforming humans on divergent-thinking tasks (Hubert et al., 2024), whereas others find top-quartile humans exceeding the best LLM outputs (Koivisto & Grassini, 2023). The field therefore lacks a structured framework for evaluating whether—and how—LLMs succeed as creative idea generators, cognitive models, and assessment tools.

**Figure 1:**
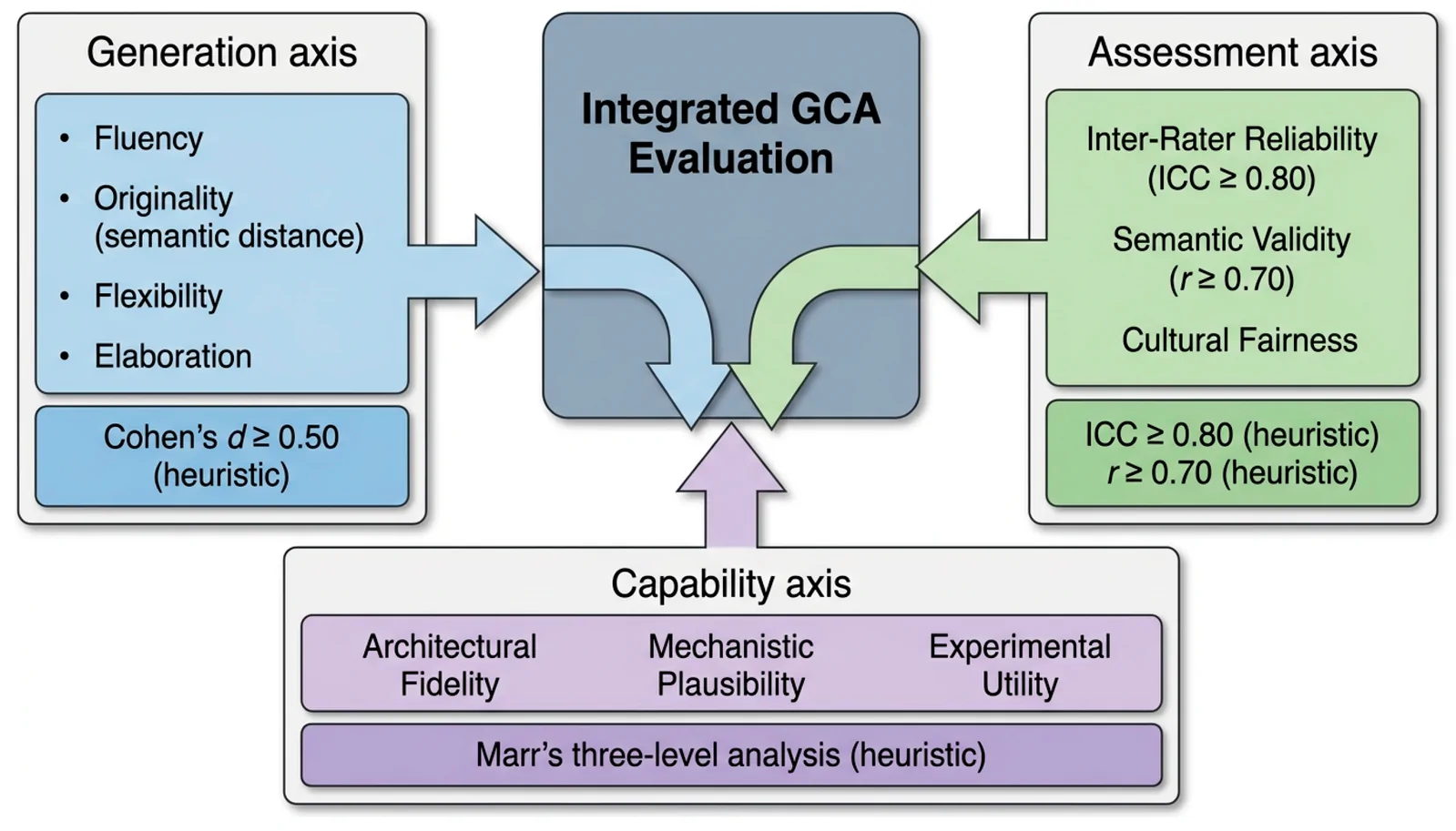
The GCA framework for evaluating LLMs in creative thinking research. Each axis displays its evaluation criteria and the corresponding provisional heuristic benchmark.

This review addresses that gap through the generation–capability–assessment (GCA) framework, a structured rubric whose three interdependent axes (Figure 1) are each operationalised through quantitative criteria with provisional heuristic thresholds. The generation axis asks whether LLM outputs meet quantitative criteria for creative quality—fluency (number of distinct responses), originality (semantic distance from prompt and population norms; heuristic threshold Cohen’s d ≥ 0.50, a medium effect by conventional benchmarks (Cohen, 1988) and consistent with the generalised gains reported in quantitative reviews of creativity training (Scott et al., 2004)), flexibility (number of conceptual categories spanned), and elaboration (level of detail)—benchmarked against human performance. The capability axis asks whether LLM architectures constitute valid cognitive models of creative thought, assessed through architectural fidelity, mechanistic plausibility, and experimental utility against Marr’s (1982) three levels of analysis (developed in Section 5.1). The assessment axis asks whether LLM-based scoring systems meet psychometric standards: inter-rater reliability (ICC ≥ 0.80, following the psychometric convention for clinical-instrument adequacy), semantic validity (r ≥ 0.70, approximately 50% shared variance with human ratings), and cultural fairness. Throughout this study, these thresholds are used as provisional descriptive benchmarks for profiling studies, not validated evaluative criteria.

The review is structured as follows. Section 2 describes the literature search and selection strategy. Section 3 establishes the theoretical foundations of creative cognition and the architectural principles of transformer-based LLMs. Section 4, Section 5 and Section 6 evaluate the evidence for the three GCA axes—LLMs as creative idea generators, as cognitive models of creative thought, and as automated assessment tools. Section 7 examines human–AI co-creativity. Section 8 addresses methodological challenges, ethical concerns, and open questions, including a dedicated limitations subsection. Section 9 synthesises the evidence and provides a practical GCA application protocol.

Before proceeding, two levels of claim must be distinguished, and this distinction is maintained throughout the review. Behavioural-performance claims concern observable output quality (semantic-distance scores, subjective ratings, and similar measures) and can be true of LLM outputs without implying anything about the processes that produced them; psychological-process claims concern mechanisms, intentionality, and understanding. Section 4 evaluates behavioural evidence and its boundary conditions, Section 5 evaluates process-level claims through the simulation–instantiation distinction, and Section 9 returns to the distinction in synthesis.

## 2. Literature Selection Strategy and Methods

We searched five databases—PubMed, PsycINFO, Web of Science, Scopus, and arXiv—for work published between January 2018 and June 2026, capturing the emergence of transformer-based LLMs from the architecture itself (Vaswani et al., 2017) through early pretrained models (Devlin et al., 2019; Brown et al., 2020) to the most recent developments. The search combined three keyword blocks—creativity terms, LLM terms, and assessment terms—joined with AND (Supplementary Table S1), with results deduplicated in Zotero (version 10.0.1).

Studies were included if they (a) explicitly employed LLMs or transformer-based language models as a primary research tool, subject, or object of study; (b) addressed at least one dimension of creativity as operationalised in the GCA framework; and (c) were published in English in peer-reviewed journals, conference proceedings, or as high-quality preprints. Studies were excluded if they (a) focused exclusively on non-LLM AI systems (e.g., traditional machine-learning or rule-based systems); (b) addressed creativity only in a metaphorical or non-operationalised sense; or (c) were editorials, commentaries, or book reviews without original empirical or theoretical contributions. The search window (January 2018–June 2026) is distinct from the broader theoretical sources (1950–2026) brought in through citation tracking (Guilford, 1950; Mednick, 1962). Two authors screened titles and abstracts independently, resolving disagreements through discussion (inter-rater agreement: κ = 0.82). Table 1 summarises the criteria and their justifications.

After deduplication and screening, 104 papers formed the final library. Of these, 79 are cited in this review on the basis of their relevance to the GCA framework; the reference list comprises 81 entries, including two foundational theoretical sources identified through citation tracking. The full library, with extraction fields (study design, GCA-axis allocation, sample, and key findings), is available from the authors upon request. Supplementary Table S2 allocates the key cited studies to the generation, capability, or assessment axis according to each study’s primary contribution.

This is a selective narrative review: the structured search and PRISMA-informed elements (Page et al., 2021) are adopted as transparency devices, not as claims of systematic-review comprehensiveness, and the GCA framework serves as an organising heuristic for a fragmented literature rather than a validated measurement instrument. Because the included studies span heterogeneous methodologies, a quantitative meta-analysis was not feasible; Section 4 (Table 2) therefore reports effect sizes study by study, and differences in task, outcome measure, and fluency-control procedure preclude a defensible pooled estimate. The review was not pre-registered (see Section 8.4).

## 3. Foundations of Creative Thinking and the Rise of LLMs

### 3.1. Theoretical Frameworks of Creative Cognition

Creativity research has converged on computationally tractable core operations. The dual-process model distinguishes divergent from convergent thinking (Guilford, 1950; Cropley, 2006; Chrysikou, 2019); Mednick’s (1962) associative theory locates individual differences in the organisation of semantic memory; the Geneplore model specifies iterative cycles of generation and exploration (Finke et al., 1992); and the Four C model and the standard novelty-plus-usefulness definition locate LLM creativity relative to human achievement (Rhodes, 1961; Runco & Jaeger, 2012; Kaufman & Beghetto, 2009; Green et al., 2024).

These frameworks converge on three core operations: associative retrieval, whose breadth determines the novelty of what is retrieved; controlled attention, which maintains goals, inhibits dominant responses, and switches categories; and iterative evaluation against task constraints. This convergence provides a principled basis for asking whether LLMs—which implement associative retrieval through attention, controlled generation through decoding strategies, and a form of iterative evaluation through prompt engineering—can serve as valid models of creative cognition (Beaty & Kenett, 2023; Kozbelt et al., 2010).

### 3.2. Neurocognitive Architecture of Human Creativity

Neuroimaging research has identified a dual-process neural architecture supporting creative thought (Benedek & Fink, 2019): the default-mode network (DMN)—medial prefrontal, posterior cingulate, and angular cortex—is active during spontaneous, self-generated thought and associative processing, while the executive-control network (ECN)—dorsolateral prefrontal and posterior parietal cortex—supports controlled attention, working memory, and goal-directed evaluation, and the salience network mediates switching between them (Beaty et al., 2018; Lloyd-Cox et al., 2022). Individual differences in creative ability are linked to structural and functional brain properties (Beaty et al., 2018), and the creativity–intelligence relationship, although modest, is meaningful (Nusbaum & Silvia, 2011).

This architecture offers a heuristic frame for the generative–evaluative dynamics of LLM output: in the neural model the DMN generates candidate ideas through spontaneous association and the ECN evaluates them, whereas in LLMs the transformer produces candidate tokens and decoding strategies modulate output diversity. The analogy is heuristic rather than exact (Section 5.1), but it provides a conceptual bridge between the neural and computational levels of analysis.

### 3.3. Transformer Architectures as Associative Engines

The self-attention mechanism at the core of transformer-based LLMs (Vaswani et al., 2017) computes learned, context-dependent weights between token representations, retrieving contextually relevant information from across the input sequence—a form of associative retrieval compared with spreading activation in human semantic memory (Kenett et al., 2014). The comparison is heuristic: transformer attention is a deterministic computation over a fixed context window, whereas human spreading activation is recurrent, stochastic, and neuromodulated (Section 5.1).

The scaling hypothesis—that larger models trained on more data develop qualitatively different capabilities—is particularly relevant: larger LLMs may develop flatter associative hierarchies in which remote associations become more accessible, mirroring the structure Mednick (1962) identified as the cognitive basis of individual differences in creativity. This structural parallel is more than metaphorical: it provides a computational mechanism through which the associative theory can be instantiated and tested at scale; how deep the comparison runs is an empirical question taken up in Section 5, where the simulation–instantiation distinction (Section 5.3) provides the evaluative framework.

## 4. LLMs as Creative Idea Generators

### 4.1. Benchmarking LLM Creativity on Standard and Alternative Tasks

The most direct test of the generation axis compares LLM outputs with human responses on standardised creativity tasks. Demszky et al. (2023) provided an early framework for such work, cautioning that LLM outputs are statistical approximations of human language rather than products of genuine creative thought. Hubert et al. (2024) compared GPT-4 with 151 human participants on the Alternative Uses Task (AUT), the Consequences Task, and the Divergent Association Task, and found GPT-4 to be “robustly more creative”—a behavioural-level description of output quality—on every divergent-thinking measure, including after fluency matching. Two qualifications apply: the matching was post hoc (the LLM generated exhaustively and its responses were down-sampled to each participant’s response count), so the comparison does not equate the generative search spaces, with consequences examined in Section 4.5; the classic tasks predate current LLMs, with unknown exposure status in proprietary training corpora (Section 8.1), so the corresponding effect sizes should be read as upper-bound estimates pending temporal-holdout replications.

Grassini and Koivisto (2025) compared ChatGPT-4 with human participants on the Figural Interpretation Quest (FIQ), a multimodal task requiring creative interpretation of ambiguous abstract figures. The pattern was nuanced: AI showed higher average flexibility—more semantically diverse interpretations—but humans were rated as subjectively more creative, and the most creative human responses exceeded the best AI responses on both measures. The finding matters because the FIQ engages perceptual and semantic processes beyond the purely verbal divergent thinking assessed by the AUT. The dissociation between average performance (where AI excels) and peak performance (where humans excel) implies that the generation axis must distinguish typical from exceptional creative output.

Performance also varies substantially across tasks. LLMs perform well on the AUT, whose simple prompt–response format maps naturally onto autoregressive language generation. They perform less well on tasks requiring sustained narrative coherence: Bellemare-Pepin et al. (2026) found that LLMs approach but do not match human creative-writing ability across haiku, story synopses, and flash fiction, and Vinchon et al. (2024) found that ChatGPT generated content fluently but at average rather than exceptional creativity. Breithaupt et al. (2024) found that humans create more novelty than ChatGPT when retelling a story. The LLM advantage is thus task-specific rather than domain-general, which constrains the generation axis: its criteria apply most directly to verbal divergent-thinking tasks and less directly to narrative, visual, or multimodal creativity.

Beyond laboratory tasks, recent work has evaluated LLM divergent thinking in scientific idea generation. Ruan et al. (2026) introduced LiveIdeaBench, which assesses idea generation from single-keyword prompts with minimal context—a design that deliberately removes the rich contextual scaffolding on which LLMs typically excel. Drawing on Guilford’s (1950) theory, a dynamic panel of state-of-the-art LLMs rated responses across five dimensions (originality, feasibility, fluency, flexibility, and clarity) for more than 40 leading models and 1180 keywords spanning 22 scientific domains. Notably, the scientific idea-generation capabilities measured by this benchmark were poorly predicted by general intelligence scores: models such as QwQ-32B-preview achieved creative performance comparable to frontier models despite substantial gaps in general intelligence metrics. These findings reinforce the GCA premise that creative capability is a distinct construct requiring dedicated evaluation rather than inference from general performance, and they extend the generation-axis evidence to the most recent model generation.

### 4.2. Prompt Engineering and the Controllability of Creative Output

Prompt engineering has emerged as a critical methodological variable: wording, examples, and response format substantially influence the novelty, diversity, and appropriateness of LLM output. Question-asking and prompt engineering draw on shared creative-inquiry skills (Lazovsky et al., 2025), practical prompting strategies exist for educational contexts (Park & Choo, 2024), validity-guided workflows treat prompt design as an experimental manipulation analogous to varying task instructions (Lin, 2026), and prompt sensitivity is a pervasive reliability challenge (Kostikova et al., 2026). Controlled experimental estimates of these effects on creativity outcomes remain scarce, however; claims about specific prompt or decoding variables should be treated as hypotheses until such comparisons exist.

Consequently, creative output is not a fixed property of a model but a function of the model–prompt interaction. Controllability is both a strength—it lets researchers probe the boundary conditions of LLM creativity systematically—and a limitation, because every single-study result is conditional on the prompt used. The GCA framework therefore recommends reporting prompts in full using a standardised template covering the exact prompt text, model version, temperature, and number of responses generated.

### 4.3. The Novelty–Typicality Trade-Off and Decoding Strategies

The novelty–typicality trade-off is a characteristic tension in LLM-based creative generation. LLMs are trained to maximise the likelihood of the next token given the preceding context—an objective that favours statistically typical outputs over novel ones (Bender et al., 2021). This objective sits uneasily with the definition of creativity as ideas that are both novel and useful: the most likely output is rarely the most novel, and the most novel output is rarely the most likely.

Decoding strategies modulate this trade-off: temperature tuning raises the probability of lower-likelihood tokens, increasing diversity at the cost of coherence; nucleus (top-p) sampling balances the two; and diversity-promoting objectives such as maximum mutual-information decoding optimise for diversity explicitly. The GCA rubric captures the trade-off by requiring that fluency and originality be reported together.

Convergent output compounds the trade-off: LLMs reproduce training-data patterns more extensively than previously recognised (McCoy et al., 2023), and humans sometimes prefer anomalous low-probability alternatives that LLMs systematically avoid (Brandt, 2025). Repeatedly prompted, an LLM’s outputs cluster around a small set of high-probability responses; without explicit diversity-promoting strategies, it may generate superficially different but semantically similar ideas, limiting its value as a creative collaborator.

### 4.4. Evidence Against Attributing Creativity to LLMs

The case against attributing creativity to LLMs rests on three pillars. These arguments concern psychological process rather than behavioural performance: none of them disputes that LLM outputs can score highly on behavioural measures of creative quality (Section 4.1). The first pillar is the absence of intentional content: LLMs generate text by maximising the statistical likelihood of token sequences, without internal representation of meaning or purpose (Bender et al., 2021; Demszky et al., 2023). An LLM that produces a novel word sequence has no goal, intention, or understanding of what it has generated; the output may be novel but is not creative in the psychological sense. This “stochastic parrots” argument (Bender et al., 2021) holds that LLM outputs are permutations of patterns learned from human text; in Boden’s (2004) terms, current LLM output appears primarily combinational, with limited exploratory capacity and negligible evidence of transformational creativity.

The second pillar is the lack of conscious evaluation: human creative thought involves evaluating candidate ideas against internal standards of quality and novelty, whereas LLM outputs are the direct product of a decoding strategy applied to a probability distribution; even the appearance of self-critique is itself a generated token sequence, not a genuine evaluative process.

The third pillar is disembodiment: human creativity is shaped by embodied experience, emotional engagement, and social context (Glăveanu, 2014), and LLMs trained on text lack direct experience of the world, limiting output in domains requiring embodied knowledge—novel physical objects, emotionally grounded music, or hypotheses grounded in direct observation of nature.

Recent evidence complicates attribution further: in human–AI collaborative problem-solving, the quality of social interaction rather than the perceived cognitive utility of the AI predicted positive affective experiences, and this affective pathway was linked specifically to convergent rather than divergent thinking (Y. Cheng & Huang, 2026). LLMs thus cannot participate in the affective and social dimensions of collaboration that are constitutive of human co-creativity.

Whether raters can discriminate AI-generated from human-generated creative responses is a question adjacent to attribution. Studies of attributed authorship (Stanko-Kaczmarek et al., 2025) and of bias against AI creativity (Magni et al., 2024) have examined whether people evaluate creative products differently when they believe them to be AI-generated. But the direct question—whether raters can tell AI from human creative responses above chance—has not been the primary target of any study included in this review. The GCA framework identifies this gap as a priority for future research.

### 4.5. Contradictory Findings and Boundary Conditions

The generation-axis literature is characterised by three systematic contradictions, which together define its boundary conditions. The first is the fluency dependency of the LLM advantage. When LLMs generate exhaustively—dozens of responses to a single prompt—the most original of these often exceeds the originality of the average human response, and the magnitude of the advantage tracks the stringency of fluency control. Under prospective matching, in which the AI was constrained in advance to produce the same number of responses as human participants, the originality advantage was moderate (Koivisto & Grassini, 2023). Under post hoc matching, in which the LLM first generated exhaustively and its outputs were subsequently down-sampled to human response counts, the advantage was several times larger (Hubert et al., 2024). Because task, scoring, and sample also differ between these two studies, this contrast is consistent with fluency dependency but not diagnostic of it. The GCA rubric addresses the confound by requiring that fluency be reported alongside originality and that LLM–human comparisons control for the number of responses.

The second contradiction concerns peak human performance. Koivisto and Grassini (2023) observed that the best human responses exceeded the best AI outputs under the most stringent criteria, although the corresponding max-score comparisons were statistically non-significant (Table S2). The ceiling is better established at scale: Bellemare-Pepin et al. (2026) benchmarked state-of-the-art LLMs against 100,000 human participants using identical, objective semantic-divergence scoring across the Divergent Association Task and several creative-writing tasks (haiku, story synopses, and flash fiction). The best-performing model (GPT-4) exceeded the population-average score but did not exceed the mean of the top 50% of human participants (Table 2), and on the figural FIQ the most creative human sessions likewise exceeded the best AI sessions (Grassini & Koivisto, 2025). That ceiling may reflect an architectural limitation: LLMs are optimised to model the central tendency of the training distribution, whereas the most creative human responses are, by definition, statistical outliers. The GCA generation-axis threshold (d ≥ 0.50) is calibrated to the average human comparison, not the peak one; researchers should report both when claiming LLM creative superiority.

The third contradiction is cross-task variability (Section 4.1): performance on the AUT does not consistently predict performance on the TTCT, creative story generation, or real-world problem-solving (Vinchon et al., 2024; Breithaupt et al., 2024), and the variability extends to scientific idea generation, where benchmark performance is poorly predicted by general intelligence scores (Ruan et al., 2026). The GCA framework therefore recommends multi-task evaluation with task-specific reporting rather than reliance on any single task as a proxy for general creative ability.

**Figure 2:**
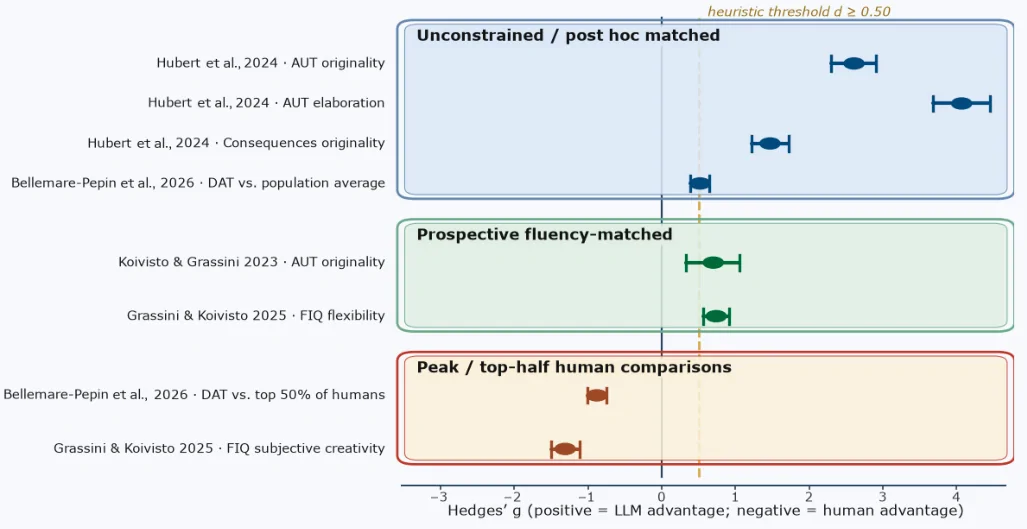
Boundary conditions of the LLM creative advantage. Forest plot of Hedges’ g (95% CI) for the three comparison levels: unconstrained or post hoc matched comparisons (largest LLM advantage), prospectively fluency-matched and other independent comparisons (moderate advantage), and peak/top-half human comparisons (human advantage). Positive values favour LLMs; values are taken from Table 2. (Hubert et al., 2024; Koivisto & Grassini, 2023; Grassini & Koivisto, 2025; Bellemare-Pepin et al., 2026).

Figure 2 summarises these comparison levels as a forest plot of Hedges’ g (values from Table 2); together, the three levels define the boundary conditions of the generation axis.

Across the four independent comparisons, effect sizes favouring LLMs over average human performance range from g = 0.52 to g = 2.61, and all exceed the GCA heuristic threshold of d ≥ 0.50. The magnitude tracks task and design: the largest estimate comes from post hoc fluency matching on verbal originality (Hubert et al., 2024), prospective matching (Koivisto & Grassini, 2023) and figural flexibility (Grassini & Koivisto, 2025) yield moderate estimates, and the large-scale DAT comparison (Bellemare-Pepin et al., 2026) yields the smallest. Peak-level comparisons run in the opposite direction (g = −0.88 against the top 50% of 100,000 participants; g = −1.31 for figural subjective creativity). Given the different tasks, measures, and fluency-control procedures, these estimates are separate data points rather than a single pooled effect.

In summary, the LLM advantage on the generation axis is real but conditional—on the task, the measure, and the fluency-control procedure; the value of the GCA framework lies in making those conditions explicit.

## 5. LLMs as Cognitive Models of Creative Thought

### 5.1. Marr’s Levels Applied to LLM Creativity

Marr’s (1982) three-level framework offers a principled structure for evaluating whether LLMs constitute valid cognitive models of creativity. At the computational level, LLMs and human creative cognition address the same problem: generating contextually appropriate, sufficiently novel responses to an open-ended prompt. At the algorithmic level, LLMs implement associative retrieval through self-attention and controlled generation through decoding strategies, paralleling spreading activation in semantic memory (Mednick, 1962; Kenett et al., 2014) and executive-control modulation of default-mode network output (Beaty et al., 2018).

At the implementation level, the gap is fundamental: transformer attention is a deterministic, feedforward computation over a fixed context window, whereas biological neural computation is recurrent, stochastic, and neuromodulated. The GCA capability-axis rubric therefore evaluates each level independently; claims about computational or algorithmic modelling do not require implementation-level correspondence.

### 5.2. Empirical Evidence for and Against LLMs as Cognitive Models

The empirical evidence is mixed. Positively, LLMs replicate associative phenomena central to the associative theory: the flattening of the associative hierarchy with model scale (Section 3.3) mirrors the individual-difference variable Mednick (1962) identified as the cognitive basis of creativity, and the context-dependence of LLM semantic access parallels the context dependence of human semantic retrieval (Beaty & Kenett, 2023; He et al., 2021).

Negatively, LLMs fail to replicate insight—the sudden realisation of a solution accompanied by surprise, whose neural mechanisms have no counterpart in LLMs (Aru et al., 2023)—incubation effects, and the conscious evaluative dimension of creative thought. These failures suggest that LLMs are best viewed as partial models of the associative component of creativity, not comprehensive models of the creative process (Beaty & Kenett, 2023; Mahowald et al., 2024; Fazi, 2018).

The individual-difference evidence is particularly instructive: human divergent-thinking performance correlates with openness to experience, working-memory capacity, and semantic-memory structure (Silvia et al., 2008; Nusbaum & Silvia, 2011; Perchtold-Stefan et al., 2020), and attempts to map these variables onto LLM behaviour have produced inconsistent results, suggesting that the mechanisms underlying LLM output are not isomorphic to those underlying human creative output. Mahowald et al. (2024) distinguish formal from functional competence in LLMs, and X. Wang et al. (2024) showed that semantic associative abilities and executive control interact in predicting human creativity—an interaction not yet demonstrated in LLMs.

In summary, the capability-axis evidence indicates that LLMs are useful but partial cognitive models of creative cognition: they replicate associative phenomena at the computational and algorithmic levels but fail to capture insight, incubation, evaluation, and individual-difference patterns.

### 5.3. Simulation Versus Instantiation: A Methodological Framework

The simulation–instantiation distinction is central to the capability axis. Simulation occurs when an LLM produces outputs resembling those of creative humans without its internal mechanisms being analogous to human cognitive processes. Instantiation occurs when an LLM operates through mechanisms that are meaningfully analogous to the processes posited by psychological theories of creativity.

The simulation claim is methodologically defensible and useful: when an LLM’s output patterns replicate known psychological phenomena—such as the relationship between associative hierarchy and originality—the model can serve as a computational testbed for exploring boundary conditions and generating hypotheses for human studies (Zhao et al., 2025). GPT-3 tested on the AUT shows both convergences with and divergences from human performance (Stevenson et al., 2022), LLM decision-making resembles human behaviour in predictable ways (Han et al., 2024), and a “sweet spot” for creative ideation—non-linear associations between semantic distance and creativity—parallels the inverted-U relationship proposed by associative theory (Orwig et al., 2025).

The instantiation claim requires evidence that the internal computations of LLMs map onto the cognitive processes posited by psychological theories. Such evidence is currently lacking: LLM representations are vectors in a high-dimensional embedding space whose relationship to psychological constructs is not well understood. The GCA rubric therefore recommends a simulation-default stance, with instantiation claims advanced only when accompanied by explicit evidence of mechanistic correspondence. Such claims should specify the level of Marr’s (1982) analysis at which correspondence is claimed—computational, algorithmic, or implementation—and the evidence supporting it at that level.

## 6. LLMs as Automated Creativity Assessment Tools

### 6.1. Semantic Distance as a Computational Proxy for Originality

The theoretical foundation of automated creativity scoring lies in the associative theory of creativity (Mednick, 1962): if creativity is mediated by the organisation of semantic memory, originality can be quantified as semantic distance from the prompt, from other responses, or from population norms. Transformer-based embeddings—context-dependent vectors in a high-dimensional space—have largely replaced latent semantic analysis for automated scoring (Beaty et al., 2022; Organisciak et al., 2023; Beaty & Johnson, 2021).

Convergent validity comes from moderate-to-strong correlations between semantic-distance scores and human originality ratings (Beaty et al., 2022; Organisciak et al., 2023); discriminant validity from stronger correlations with originality than with fluency or elaboration (Beaty et al., 2022); and criterion validity from prediction of external criteria such as creative personality and expert evaluations (Beaty et al., 2022; Orwig et al., 2021; Liu et al., 2021). The GCA rubric operationalises semantic validity through the heuristic criterion r ≥ 0.70 for group-level research; because correlation indexes association rather than agreement, it does not establish interchangeability with human ratings, and individual-level assessment would require decision accuracy, calibration, and person-level error analysis that the current literature does not support. Orwig et al. (2024) extended this work to the language of creativity in both humans and LLMs.

### 6.2. Automated Scoring Systems for Divergent Thinking

The ecosystem of LLM-based scoring tools has expanded rapidly: Organisciak et al. (2023) showed that LLM-based scoring substantially improves on traditional semantic-distance approaches, achieving intraclass correlations above 0.80 against expert ratings; OCSAI (Organisciak’s Computational Scoring of Alternate Uses and Ideation), developed by Organisciak and colleagues, combines semantic-distance computation with LLM-based judgement to produce originality, flexibility, and elaboration scores approaching trained-rater reliability; Saretzki and Benedek (2026) provided independent validity evidence on cross-language generalisation with calibration; and Brickman et al. (2025) reviewed both the promise and the psychometric challenges.

Automated scoring has also expanded beyond English: CreaScorer for scientific creativity in German (Goecke et al., 2024) and TransDis for Chinese divergent thinking (Yang et al., 2023) show that the approach generalises when the embedding model is trained on the target language. The GCA rubric evaluates scoring systems on inter-rater reliability (ICC ≥ 0.80), semantic validity (r ≥ 0.70), cultural fairness, and scalability; current tools show promising reliability and validity for group-level analysis in specific languages, but cultural fairness remains a significant challenge (Section 6.3). Reported ICC values also differ in model form (one-way vs. two-way), unit, and target, and most primary studies do not specify these parameters; the GCA framework recommends reporting the ICC form, confidence intervals, and repeatability conditions explicitly.

In summary, LLM-based scoring tools approach the heuristic thresholds for reliability and validity in their training language, but the cross-linguistic reliability gap and domain-specific calibration remain barriers to widespread deployment (Section 6.3).

### 6.3. Cross-Cultural Validity and the Cross-Linguistic Reliability Gap

LLM-based assessment tools may face a cross-linguistic reliability gap, although paired within-tool comparisons across languages are largely absent: applied outside their training language, agreement with human raters has tended to be lower and semantic-validity correlations weaker (Yang et al., 2023; Guo et al., 2024). The gap arises because embedding models trained on predominantly English corpora learn the distributional properties of English text, so computed distances may not capture culturally specific dimensions of creativity; Guo et al. (2024) found scalar invariance for fluency but only partial scalar invariance for originality between American and Chinese students.

This gap has direct implications for cross-cultural deployment. In educational contexts, where creativity assessment increasingly informs admissions, placement, and evaluation, a tool that is valid in one language but not another introduces systematic bias. The GCA rubric therefore requires researchers to report the language of the embedding model used and, when a tool is applied outside its training language, to provide evidence of language-specific calibration (Yang et al., 2023; Goecke et al., 2024). Bearman et al. (2024) similarly argue that human oversight remains essential for valid creativity assessment in the era of generative AI.

Beyond embedding-model limitations, cross-cultural validity raises construct-level concerns. Conceptions of what counts as novel and useful vary across cultures, and originality-as-semantic-distance operationalises novelty relative to the distributional norms of the embedding corpus—predominantly English-language text produced by Western-educated authors. A tool that scores deviation from those norms may therefore conflate cultural difference with lower creativity, imposing one culture’s standards on another. The partial scalar invariance reported above is direct evidence that originality scores are not culturally interchangeable. The GCA cultural-fairness criterion accordingly requires evidence of construct equivalence—not merely comparable reliability or validity coefficients—before cross-cultural comparisons are made, and Section 8.2 treats this as an ethical as well as a psychometric requirement.

Narrative and artistic creativity pose additional challenges: extended, multimodal outputs cannot be captured by a single semantic-distance score, and hybrid approaches combining computational metrics with human judgement are recommended (Vinchon et al., 2024). The GCA criteria accordingly require human ratings for at least a subset of such outputs, with the automated–human correlation reported as a validity indicator. A further constraint is domain specificity: creativity varies across domains (Baer, 2012; Kaufman & Baer, 2004), so tools validated on verbal divergent thinking may not generalise without domain-specific calibration.

In summary, LLM-based scoring tools are reliable and valid for English-language group-level analysis, but cross-language use requires language-specific calibration. Cross-language generalisation and cultural fairness remain largely untested; the gaps described here should be read as an absence of evidence rather than a demonstrated systematic decline, and they constitute the most significant unmet criterion of the GCA assessment axis.

## 7. Human–AI Co-Creativity and Collaborative Ideation

### 7.1. Mechanisms of Effective Human–AI Creative Collaboration

The co-creativity paradigm shifts the focus to LLMs as partners. A commonly proposed model involves a division of labour: AI expands the generative search space and humans apply evaluative judgement and domain knowledge (Koivisto & Grassini, 2023). ChatGPT assistance increased idea creativity relative to unaided thinking and web search, with larger benefits for incremental than for radical innovation (Lee & Chung, 2024), and LLM ideation partners increased idea quantity and final-design quality provided humans retained evaluative authority (P. Wang et al., 2026). Not all comparisons favour AI: human–human dyads outperformed human–AI dyads on divergent thinking and uniquely boosted creative self-efficacy (Tang et al., 2025), and generative AI enhanced individual creativity while reducing collective diversity (Doshi & Hauser, 2024). Importantly, none of these studies directly compared the AI-generator/human-evaluator configuration with co-equal or passive-AI alternatives; the relative effectiveness of role configurations remains an open empirical question.

The mechanisms are more nuanced than a simple division of labour: the perceived cognitive support provided by an LLM partner did not directly predict creative performance, whereas the quality of social interaction—engagement, rapport, collaborative satisfaction—was the strongest self-report correlate of positive affective experience, associated specifically with convergent rather than divergent outcomes (Y. Cheng & Huang, 2026). How the collaboration is experienced may be as consequential as what the AI generates.

The COFI framework (Rezwana & Maher, 2023) offers a structured design approach through three dimensions: the interaction, contribution, and adaptation models. Within the GCA framework, co-creativity is treated as a cross-axis application domain rather than a fourth axis: its assessment extends the generation-axis criteria to interaction quality (ideation efficiency, idea diversity, participant satisfaction).

Collaborations tend to be more productive under this AI-generator/human-evaluator configuration than under co-equal or passive-AI alternatives, and effectiveness may depend on the type of creative thinking targeted: divergent thinking may benefit from the AI’s capacity to generate large, diverse candidate sets, whereas convergent thinking may benefit more from interactions that foster social engagement and affective positivity (Y. Cheng & Huang, 2026). Group-level evidence is emerging: dyad–AI collaboration examined with fNIRS hyperscanning found that high AI-idea divergence enhanced the novelty of group ideas for everyday tasks but reduced usefulness for both everyday and scientific tasks—a novelty–usefulness trade-off mediated by AI-invocation strategies and the semantic characteristics of human ideas, with preliminary neurocognitive correlates (Zhou et al., 2026). Because the design lacked neural control conditions, shared-stimulus and coordination effects cannot be separated from creativity-specific mechanisms.

**Figure 3:**
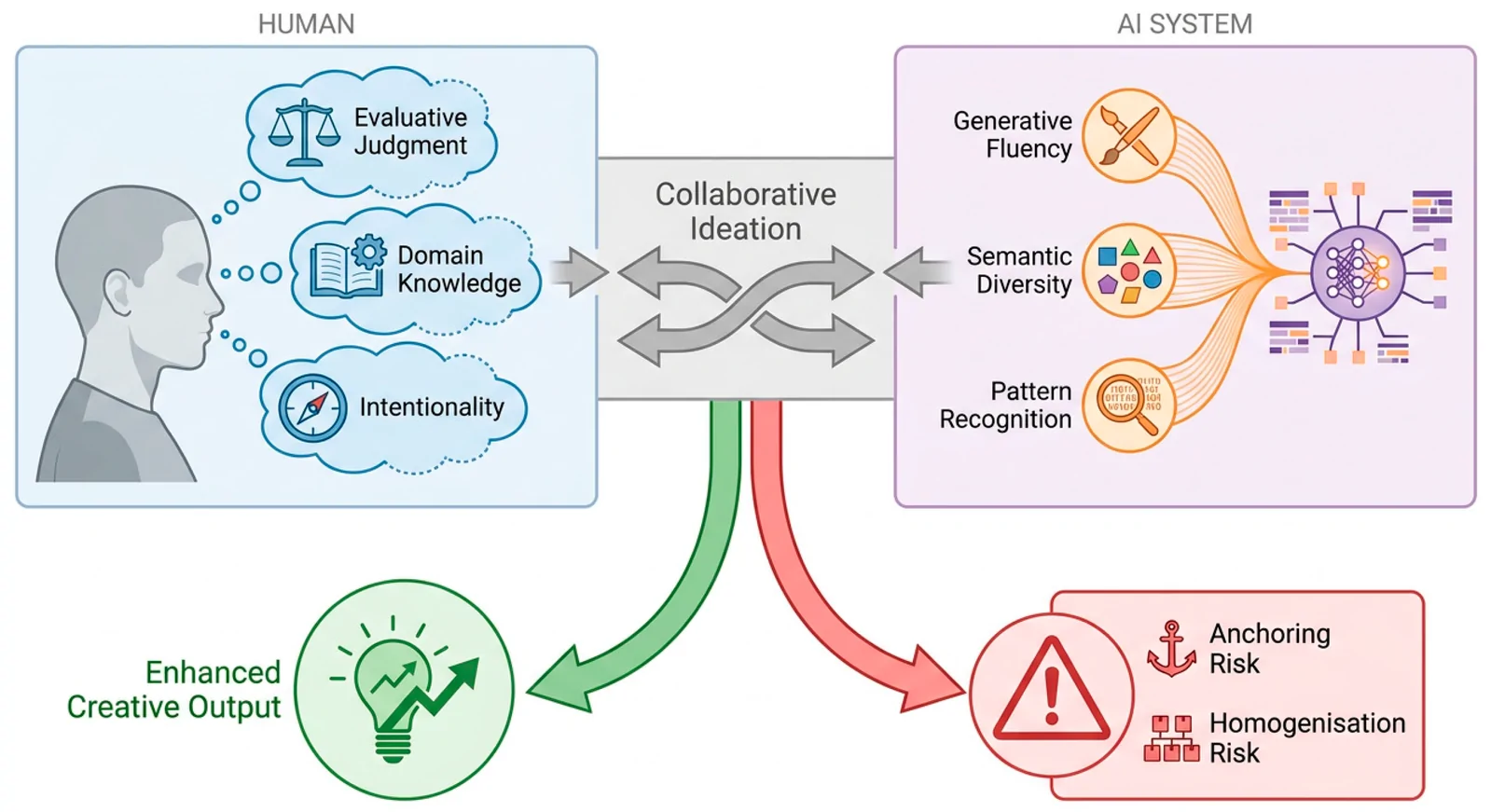
Human–AI co-creativity dynamics. The complementary strengths of human evaluative judgement and AI generative fluency converge in co-creative output, while the anchoring risk pathway illustrates the potential for AI suggestions to narrow human ideation. The anchoring-risk pathways operate at four levels of analysis (Section 7.2): evaluation, within-individual, within-model, and collective; the synthesis is qualitative, not an estimated path model. In the diagram, blue elements represent human contributions (evaluative judgement, domain knowledge, and intentionality), orange and purple elements represent AI-system contributions (generative fluency, semantic diversity, and pattern recognition), grey bidirectional arrows represent the iterative exchange of collaborative ideation, the green pathway represents enhanced creative output, and the red pathway represents the anchoring and homogenisation risks. This diagram is an original schematic prepared for this review.

Figure 3 illustrates the human–AI co-creativity dynamics, showing the complementary roles of human evaluative judgement and AI generative fluency, the feedback loop between them, and the anchoring risk pathway.

### 7.2. Anchoring Effects and the Homogenisation Risk

AI-generated suggestions can act as cognitive anchors that narrow subsequently generated human ideas, while humans are systematically biased against AI creativity (Magni et al., 2024): participants exposed to AI ideas produce responses more similar to the suggestions and less diverse (Lou & Sun, 2026); an unconstrained LLM partner improved individual creativity on simple tasks via inspiration but decreased it on complex tasks via creative fixation (X. Cheng & Zhang, 2025); and anchoring bias operates within LLMs themselves through prompt order and framing (Lou & Sun, 2026). At the collective level, generative AI can enhance individual creativity while reducing the diversity of novel content (Doshi & Hauser, 2024), and widespread deployment of AI ideation tools could homogenise creative output across cohorts (Wingström et al., 2024). These phenomena operate at different levels of analysis—attribution bias in evaluation, fixation within individuals, prompt-order effects within models, and diversity loss across outputs—and the evidence does not establish a single anchoring mechanism linking them.

The anchoring risk (Figure 3) is particularly salient in educational and professional contexts: students using AI as an ideation partner may inadvertently narrow their creative range, and teams relying on AI suggestions may converge on fewer solutions than teams working independently. The GCA co-creativity criteria therefore include a diversity-preservation metric—the semantic spread of human-generated ideas before and after AI exposure.

These risks do not negate the value of AI as a co-creative partner, but they highlight the importance of designs that preserve human creative agency. Candidate mitigations include delaying AI input until after human ideation, presenting AI ideas in randomised order, and instructing participants to treat AI suggestions as starting points; constraining LLM output mitigated fixation on complex tasks but weakened the inspiration benefit on simple tasks, so mitigations should be matched to task complexity (X. Cheng & Zhang, 2025). The GCA framework recommends testing these strategies empirically and reporting the results alongside co-creativity metrics.

In summary, human–AI co-creativity may benefit from a division of labour in which AI expands generative capacity and humans apply evaluative judgement, but the social-affective dimensions of the interaction may matter more for convergent thinking than cognitive support alone. The anchoring and homogenisation risks of AI ideation tools require design strategies that preserve human creative agency.

## 8. Methodological Challenges, Ethical Concerns, and Open Questions

### 8.1. Training-Data Contamination and Benchmark Validity

Training-data contamination is the most significant methodological challenge facing LLM creativity research. LLMs are trained on corpora that include much of the public internet, which may contain the very creativity tasks, benchmarks, and scoring rubrics used to evaluate them (McCoy et al., 2023; Bender et al., 2021). An LLM exposed to the AUT, its responses, or its scoring criteria during training may perform through memorisation rather than generalisation; the same concern applies to the TTCT, the Consequences Task, and other widely used measures.

The GCA framework therefore recommends a role-specific exposure-risk assessment rather than a binary contamination gate, with four complementary safeguards. First, pre-registration of hypotheses, methods, and analysis plans before conducting LLM creativity experiments, following the validity-guided workflow of Lin (2026). Second, temporal holdout validation: testing LLMs on tasks whose prompts and stimuli were created after the model’s training cutoff, so the specific items could not have been encountered during training. Third, dynamically generated stimuli—task items created programmatically rather than drawn from a fixed set—wherever possible. Fourth, community-maintained exclusion lists cataloguing the creativity tasks and benchmarks known to be present in LLM training data.

Contamination also interacts with benchmark validity. For open-source models, exposure risk is at least partially verifiable: training-corpus documentation, deduplication disclosures, and membership-inference probes can bound the risk, although they rarely eliminate it. For proprietary systems—spanning pretraining, instruction tuning, preference data, and model updates—proof of non-exposure is generally unavailable, and the GCA framework treats exposure as a graded risk: low when the stimuli postdate the training cutoff and the corpus is documented; moderate when the corpus is documented but the stimuli predate the cutoff; and high when the corpus is undisclosed. Classic tasks such as the AUT and TTCT predate all current LLMs, and their exposure status in proprietary corpora is unknown; for proprietary models, such comparisons therefore default to high risk, and the corresponding conclusions—including the AUT-based effect sizes in Table 2—should be read as upper-bound estimates subject to that qualification. Temporal holdout and dynamically generated items reduce exact-item exposure but do not exclude exposure to templates or close variants, and they require their own human calibration.

### 8.2. Ethics of AI Creativity: Authorship, Bias, and Environmental Costs

The ethical deployment of LLMs in creativity research involves three interrelated concerns. The first is authorship attribution: who—or what—should be credited for AI-generated content remains unresolved (Demszky et al., 2023). The GCA framework recommends differentiated disclosure standards distinguishing AI-assisted content (substantive human intellectual contribution) from AI-generated content, with the nature and extent of the AI contribution disclosed in the methods section; the aesthetic value of AI-generated content does not itself resolve creative attribution (Xu et al., 2025).

The second concern is cultural and linguistic bias: LLM-based assessment tools trained predominantly on English corpora may systematically misrepresent the creative abilities of people from non-English-speaking or non-Western backgrounds (Guo et al., 2024). The cross-linguistic reliability gap (Section 6.3) is both a psychometric and an ethical problem. The GCA framework recommends mandatory reporting of cultural-validity metrics—including the ICC and semantic-validity correlation for each cultural group separately—for tools deployed in non-Western contexts; group-specific correlations are necessary but not sufficient evidence of fairness, which also requires evidence of calibration, construct equivalence (Section 6.3), and the absence of systematic bias.

The third concern is environmental sustainability: training and deploying large LLMs consumes substantial computational resources and energy (Bender et al., 2021). The GCA framework recommends that proposals involving large-scale deployment include an environmental cost–benefit consideration—while noting that the functional units and comparators for such analyses remain to be defined—and that smaller models be preferred for routine assessment when psychometric properties are comparable.

### 8.3. Future Directions: Intentionality, Evaluation, and Ecological Validity

The next generation of LLM–creativity research must move from demonstrating creative output to explaining its mechanisms. The first priority is computational models of intentional evaluation that go beyond token prediction—for instance dual-process architectures separating generative and evaluative components, or reinforcement learning from human feedback applied to creativity criteria—while recognising that such mechanisms could improve functional critique without thereby demonstrating intentional evaluation.

The second priority is ecologically valid benchmarks capturing domain-specific creative activity: sustained creative production (e.g., composing a short story, designing an experiment), multimodal tasks (Antony & Huang, 2024)—building on early demonstrations that generative models can produce art judged novel by human evaluators (Elgammal et al., 2017)—and collaborative tasks with human partners.

A third priority is benchmarks that separately assess divergent and convergent thinking in human–AI collaboration, whose affective and social mechanisms differ systematically (Y. Cheng & Huang, 2026); a fourth is the systematic variation in model architecture, training data, and decoding parameters to test necessary and sufficient conditions for creative behaviour. Open-source models (LLaMA, Mistral, Falcon) would let researchers vary model size, data composition, and decoding parameters systematically, enabling stronger—though still incomplete, given confounded dimensions across models—causal inference about the factors contributing to LLM creative performance.

### 8.4. Limitations of the Present Review

Several limitations qualify the conclusions of this review. First, the quantitative evidence base is thin: Table 2 contains eight rows drawn from four primary studies, several of them non-independent comparisons from the same participant samples. Because the independent comparisons differ in task, outcome measure, and fluency-control procedure, no pooled effect size is reported, and each estimate must be interpreted with caution. The GCA generation-axis heuristic threshold of d ≥ 0.50 is a provisional benchmark that may be revised as the evidence base expands. Relatedly, the heuristic thresholds were informed by the same small evidence base they are used to profile; this circularity means the thresholds should be treated as descriptive anchors pending pre-registered validation rather than as criterion-referenced standards.

Second, the non-independence of data points qualifies the quantitative presentation. Several effect sizes in Table 2 come from the same participant samples, so the effective number of independent data points is smaller than the number of rows suggests; the synthesis in Section 4 is accordingly narrative rather than meta-analytic.

Third, the evidence is scoped to a specific model generation. The studies reviewed in Section 4, Section 5 and Section 6 predominantly concern GPT-3.5, GPT-4, Claude-3, and LLaMA-2, and the fluency-matching and convergent-output findings may not transfer to models with explicit inference-time reasoning procedures, such as chain-of-thought prompting or retrieval-augmented generation. The GCA evaluation should be considered specific to the model generation studied and updated as new architectures emerge.

Fourth, the rapid pace of AI development means that some results may already reflect a past stage of model capability. The findings reviewed here are a snapshot of a moving target: the specific effect sizes and performance comparisons in Table 2 may not generalise to the next model generation. This limitation is inherent to reviewing a fast-moving field, and the GCA framework is designed to accommodate updates as new evidence appears.

Fifth, the review was not pre-registered. The GCA framework was developed in response to patterns observed in the literature, and its criteria and thresholds were not specified before the search was conducted, which limits the confirmatory value of the findings and introduces the possibility of post hoc rationalisation. Future updates should be pre-registered, with criteria and thresholds specified in advance.

Sixth, the final library comprised 104 studies; 79 library studies are cited in this review, and the reference list (81 entries) includes two foundational theoretical sources identified through citation tracking. The methodological diversity of the library—experimental psychology, computational linguistics, machine learning, and cognitive neuroscience—is both a strength (it captures the breadth of the field) and a limitation (it restricts comparability across studies). Supplementary Tables S1–S4 and Figure S1 accompany this submission and will be made publicly available upon publication. Finally, the database search required creativity, LLM, and assessment or interaction terms to co-occur (AND logic across the three blocks), which may underrepresent studies addressing only the generation or capability axes; citation tracking mitigated but did not eliminate this structural limitation, and the selective approach may underrepresent recent preprints. The findings should therefore be read as a structured map of the retrievable literature, not a complete census.

## 9. Conclusions

The GCA framework reveals that LLMs currently occupy an intermediate position in creativity research. On the generation axis, LLMs reliably meet the fluency criterion and exceed average human originality across all four independent comparisons (Hedges’ g = 0.52–2.61, reported study by study rather than pooled), an advantage qualified by fluency dependency, the superiority of top-performing humans at scale, and the novelty–typicality trade-off.

On the capability axis—which carries equal weight because mechanistic correspondence is logically prior to output quality—LLMs serve as partial, computationally tractable models of the associative component of creative cognition. Parallels at Marr’s (1982) computational and algorithmic levels remain hypotheses pending direct representational analyses, causal interventions, and matched human–model manipulations, and the implementation-level gap is fundamental. LLMs thus simulate creative output in ways useful for theory testing, but evidence that they instantiate human-like cognitive processes is lacking.

On the assessment axis, scoring systems approach the heuristic thresholds for reliability (ICC ≥ 0.80) and validity (r ≥ 0.70) in their training language, with a substantial scalability advantage, but valid cross-cultural deployment requires language-specific calibration, and individual-level or high-stakes use is unsupported.

The three axes are interdependent—advances in generation require valid assessment tools, advances in assessment require generative models, and advances in capability require both—so the most significant discoveries will emerge from their intersection rather than from any axis in isolation.

For researchers applying the GCA framework, we recommend the following protocol. First, select the axis or axes relevant to the research question and apply the corresponding criteria and heuristic thresholds (Section 1; Table 3). Second, report findings as descriptive profiling—axis by axis, without aggregating into an overall judgement, for which aggregation rules and decision-validity evidence have not been established. Third, report the GCA-axis allocation of all cited studies. Table 3 translates this protocol into a design-specific application checklist.

The GCA framework is not a final answer but a provisional scaffold; its criteria and thresholds should be refined through pre-registered studies, meta-analytic updates, and community consensus. Its value lies in organising a fragmented literature, reconciling conflicting findings, and guiding the field towards a more systematic, cumulative science of LLM-based creativity research.

## Figures and Tables

**Table 1:** Inclusion and exclusion criteria.

Criterion	Type	Justification
LLMs as primary tool, subject, or object	Inclusion	Ensures relevance to LLM–creativity nexus
Addresses ≥1 GCA dimension	Inclusion	Aligns with the review’s evaluative framework
Published in English, 2018–2026	Inclusion	Captures the transformer era and ensures accessibility
Non-LLM AI systems only	Exclusion	Outside the scope of LLM-specific evaluation
Metaphorical creativity	Exclusion	Lacks operationalised creativity measurement
Editorials, commentaries, book reviews	Exclusion	No original empirical or theoretical contribution

**Table 2:** Effect sizes from LLM–human comparisons on standardised creativity tasks.

Study	Task	Measure	g	95% CI	Note
Hubert et al. (2024)	AUT	Originality	2.61	[2.30, 2.91]	Independent; post hoc fluency matching
Hubert et al. (2024)	AUT	Elaboration	4.07	[3.68, 4.46]	Same sample
Hubert et al. (2024)	Consequences	Originality	1.47	[1.22, 1.72]	Same sample
Grassini and Koivisto (2025)	FIQ	Flexibility	0.74	[0.56, 0.92]	Independent
Grassini and Koivisto (2025)	FIQ	Subjective creativity	−1.31	[−1.50, −1.11]	Same sample; human advantage
Koivisto and Grassini (2023)	AUT	Originality	0.70	[0.33, 1.06]	Independent; prospective fluency matching
Bellemare-Pepin et al. (2026)	DAT	Originality vs. population average	0.52	[0.39, 0.64]	Independent; 503 model responses vs. 500 drawn from 100,000 humans
Bellemare-Pepin et al. (2026)	DAT	Originality vs. top 50% of humans	−0.88	[−1.01, −0.75]	Same sample; human advantage

Note. g = Hedges’ g (positive = LLM advantage), computed in R (version 4.6.1) at the participant or session level of analysis from each primary study’s published descriptive statistics, or from the primary study’s open raw data where published statistics were insufficient or internally inconsistent (see Supplementary Table S4 for row-level source statistics, sample sizes, conversion formulas, and Cohen’s d equivalents). Rows marked “Same sample” are non-independent comparisons from the same participants or sessions as the preceding independent row. Rows whose 95% CI excludes zero in the human direction are marked accordingly. Because the studies differ in task (verbal vs. figural vs. associative), outcome measure, and fluency-control procedure, the independent rows are not commensurate; no pooled estimate is reported, and findings are synthesised narratively. The AUT-based comparisons rely on classic tasks with unknown exposure status in proprietary training corpora (Section 8.1) and should be read as upper-bound estimates.

**Table 3:** GCA application checklist for common study designs.

Study Design	Applicable GCA Axis/Axes	Criteria to Apply (Heuristic Benchmarks)	Required Reporting Items
Generation-focused comparison (LLM vs. human)	Generation	Fluency; originality (d ≥ 0.50, heuristic); flexibility; elaboration	Full prompt; model name and version; temperature; number of responses per prompt; fluency-control procedure (prospective vs. post hoc); average-level and peak-level comparisons; task and scoring method; contamination-risk classification (Table S3)
Assessment-tool validation	Assessment	Inter-rater reliability (ICC ≥ 0.80, heuristic); semantic validity (r ≥ 0.70, heuristic); cultural fairness	ICC form, unit, and confidence interval; blinding of human raters; embedding-model language and target language; group-level vs. individual-level use claims; language-specific calibration evidence; construct-equivalence evidence for cross-cultural use
Cognitive-modelling study	Capability	Architectural fidelity; mechanistic plausibility; experimental utility (Marr’s three levels)	Level at which correspondence is claimed (computational, algorithmic, implementation); simulation vs. instantiation stance; falsifiable predictions tested
Co-creativity study	Generation + interaction quality	Ideation efficiency; idea diversity; participant satisfaction; diversity preservation	Interaction, contribution, and adaptation models; semantic spread of human ideas before and after AI exposure; task complexity; divergent vs. convergent outcomes
Training-data contamination screening (all designs)	All axes	Graded exposure risk (low/moderate/high)	Model type (open vs. proprietary); corpus documentation status; stimulus provenance (post-cutoff, dynamic, or classic); holdout or exclusion-list procedures

Note. Thresholds are provisional heuristic benchmarks for descriptive profiling, not validated criteria (Section 8.4). GCA = generation–capability–assessment; ICC = intraclass correlation coefficient.

## Data Availability

Effect sizes and study characteristics extracted from the original publications are available in Table 2 and Supplementary Table S2. The search strings used for literature identification are provided in Supplementary Table S1. No new primary data were generated. The extracted data and analysis code are available from the corresponding author upon reasonable request.

## Supplementary Materials

The following supporting information can be downloaded at https://www.mdpi.com/article/10.3390/jintelligence14090218/s1, Table S1: Complete PsycINFO search string used for the selective narrative review; Table S2: Characteristics of key studies cited in this review, organised by GCA axis; Table S3: Quality assessment criteria for included empirical studies in LLM-creativity research; Table S4: Row-level effect-size derivations for Table 2; Figure S1: Literature search and screening flow diagram (PRISMA-informed).

## Abbreviations

**Abbreviation**	**Full Form**
AI	Artificial Intelligence
AUT	Alternative Uses Task
CAN	Creative Adversarial Network
CAT	Consensual Assessment Technique
COFI	Co-Creative Framework for Interaction
DMN	Default Mode Network
ECN	Executive Control Network
FIQ	Figural Interpretation Quest
GCA	Generation–Capability–Assessment
ICC	Intraclass Correlation Coefficient
LLM	Large Language Model
OCSAI	Organisciak’s Computational Scoring of Alternate Uses and Ideation
PRISMA	Preferred Reporting Items for Systematic Reviews and Meta-Analyses
TTCT	Torrance Tests of Creative Thinking
DAT	Divergent Association Task
fNIRS	Functional Near-Infrared Spectroscopy
LSA	Latent Semantic Analysis
