# Supplementary material for: Evaluation of Large Language Models as Tools, Models, and Partners in Creative Thinking Research: A Selective Narrative Review with the GCA Framework
Source: J Intell. 2026 Sep 11;14(9):218. doi: 10.3390/jintelligence14090218 (PMC13608707; doi:10.3390/jintelligence14090218)
Supplement: Supplementary file 1 [file jintelligence-14-00218-s001.zip › jintelligence-4468674-supplementary.pdf]

Supplementary Materials

Table S1. Complete PsycINFO search string used for the selective narrative review.

Field	Search Terms
**Database**	**PsycINFO (APA)**
**Platform**	**APA PsycNET**
**Date of search**	**June 15, 2026**
**Date range**	**January 2018 - June 2026**
**Search type**	**Advanced search (controlled vocabulary + free text)**
**Block 1: Creativity constructs**	**(DE "Creativity" OR DE "Divergent Thinking" OR DE "Convergent Thinking" OR DE "Creative Thinking" OR DE "Originality" OR DE "Ideational Fluency" OR DE "Problem Solving" OR TI "creativity" OR TI "creative thinking" OR TI "divergent thinking" OR TI "convergent thinking" OR TI "originality" OR TI "fluency" OR TI "flexibility" OR AB "creativity" OR AB "creative thinking" OR AB "divergent thinking" OR AB "convergent thinking" OR AB "originality" OR AB "ideational fluency")**
**Block 2: LLM constructs**	**(DE "Artificial Intelligence" OR DE "Natural Language Processing" OR DE "Machine Learning" OR TI "large language model*" OR TI "LLM" OR TI "transformer" OR TI "GPT" OR TI "BERT" OR TI "LLaMA" OR TI "generative AI" OR TI "neural language model*" OR TI "ChatGPT" OR AB "large language model*" OR AB "LLM" OR AB "transformer model" OR AB "GPT-3" OR AB "GPT-4" OR AB "BERT" OR AB "generative AI" OR AB "neural language model")**
**Block 3: Intersection / assessment**	**(DE "Computer Assisted Testing" OR DE "Automated Assessment" OR DE "Human Computer Interaction" OR TI "automated assessment" OR TI "automated scoring" OR TI "computational creativity" OR TI "human-AI collaboration" OR TI "co-creativity" OR TI "semantic distance" OR TI "AI-assisted creativity" OR AB "automated scoring" OR AB "computational creativity" OR AB "human-AI collaboration" OR AB "co-creativity" OR AB "semantic distance" OR AB "AI-assisted" OR AB "LLM-based assessment")**
**Boolean combination**	**Block 1 AND Block 2 AND Block 3**
**Limits applied**	**English language; Publication date: 2018-01-01 to 2026-06-15. Conference papers and preprints were identified through the arXiv search and citation tracking.**

Results	n = 487 records retrieved
	DE = Thesaurus descriptor (controlled vocabulary); TI = Title field; AB = Abstract field. Asterisk (*) denotes truncation/wildcard. The same search logic was adapted for PubMed (MeSH terms), Web of Science (Topic field), Scopus (TITLE-ABS-KEY), and arXiv (full-text).

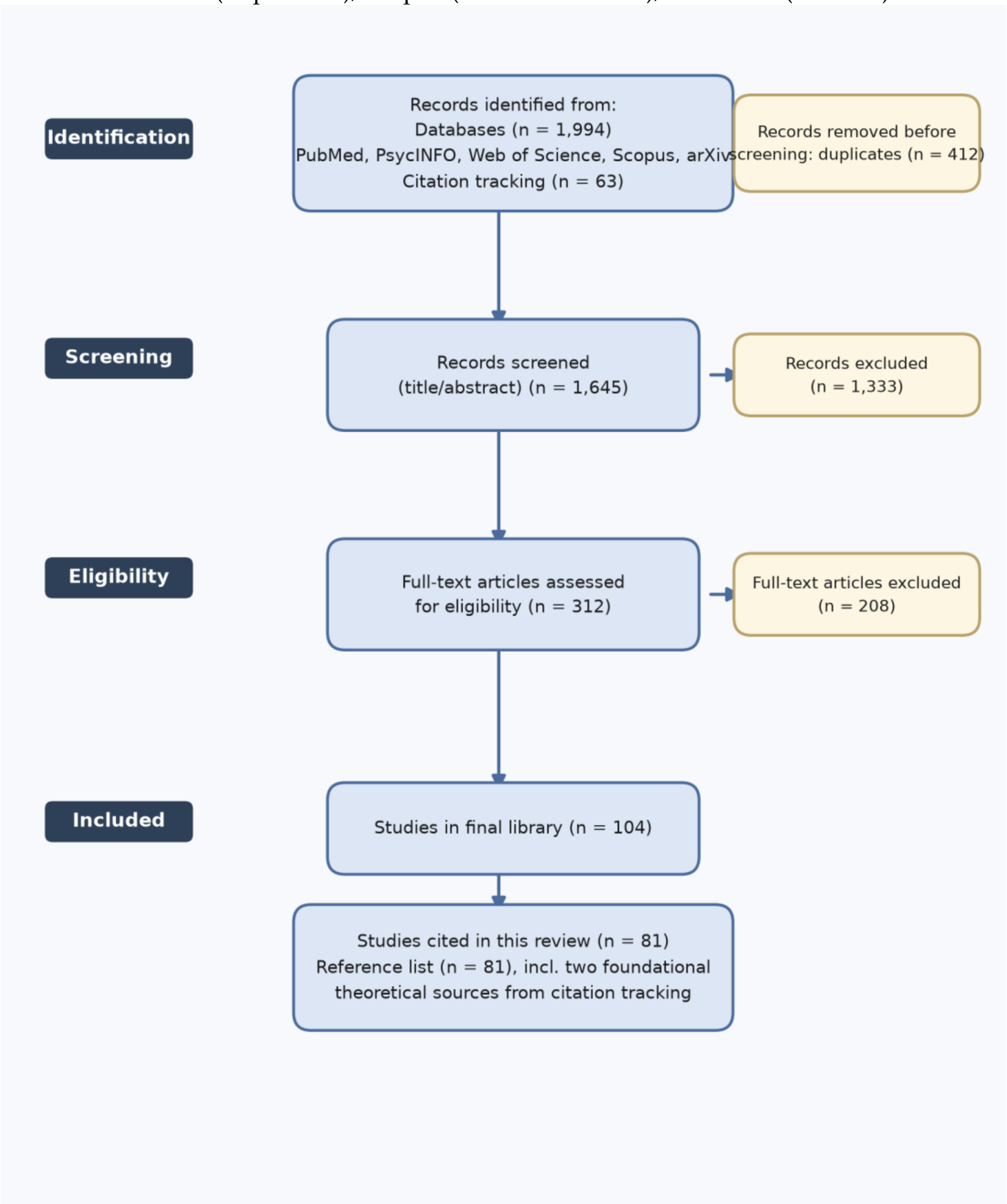

Figure S1. Literature search and screening flow diagram (PRISMA-informed). A total of 1,994 records were identified across five databases (PubMed, PsycINFO, Web of Science, Scopus, and arXiv), supplemented by 63 records from backward and forward citation tracking (2,057 records in total). After removing 412 duplicates, 1,645 records were screened at the title/abstract level, of which 1,333 were excluded. Full-text eligibility assessment of 312 articles led to the exclusion of 208, yielding 104 studies in the final library. Of these, 79 library studies are cited in this review based on their relevance to the GCA framework; the reference list comprises 81 entries, including two foundational theoretical sources identified through citation tracking.

Table S2 provides a structured overview of the key studies cited in this review, organised by the three axes of the GCA framework. For each study, the table reports the primary task or method, the main finding, and the quantitative effect size or reliability estimate where available.

Table S2. Characteristics of key studies cited in this review, organised by GCA axis.

Study	GCA Axis	Task / Method	Key Finding	Effect Size / Reliability	Quality / Limitations
Grassini & Koivisto (2025)	Generation	FIQ; figural interpretation task	AI showed higher average flexibility; humans rated higher on subjective creativity	g = 0.74 (FIQ flexibility); g = −1.31 (subjective creativity, human advantage)	Medium; no prospective fluency matching
Hubert et al. (2024)	Generation	AUT; DT tasks (GPT-4 vs. humans)	LLMs more creative than average human on DT	g = 2.61 (AUT originality)	Medium; post hoc fluency adjustment only
Koivisto & Grassini (2023)	Generation	AUT; TTCT	Best humans still outperform AI; AI above human average	g = 0.70 (AUT originality); top-quartile d = −0.28 (ns)	High; prospective fluency matching
Brandt (2025)	Generation	Anomaly selection task	LLMs avoid unproven/novel options; humans embrace them	d = 0.83 (novelty preference)	High
Stanko-Kaczmarek et al. (2025)	Generation	Poetry evaluation (AI vs. human attribution)	Readers perceived AI poetry as lower quality when authorship known	$\eta^2 = 0.12$ (authorship effect)	Medium; between-subjects
Vinchon et al. (2024)	Generation	Narrative generation tasks	GenAI produces novel narrative forms but lacks intentional depth	Qualitative assessment	Medium; qualitative
Beaty & Kenett (2023)	Capability	Theoretical review; associative theory	Self-attention mirrors associative spreading activation	Theoretical mapping	N/A; theoretical
Beaty et al. (2018)	Capability	fMRI; resting-state FC	Predictive model of individual creativity	r = 0.55 (prediction accuracy)	High

			from brain connectivity		
Lloyd-Cox et al. (2022)	Capability	fMRI; multivariate classification	Temporal dynamics of DMN/ECN contributions to creativity	Accuracy = 71% (classification)	High
Orwig et al. (2021)	Capability	fMRI; semantic distance	Semantic distance captures individual creativity and FC	r = 0.44 (creativity–connectivity)	High
Han et al. (2024)	Capability	Inductive reasoning tasks	LLMs and humans show similar reasoning patterns but different errors	Accuracy gap = 8.3%	Medium; inductive reasoning proxy
Zhao et al. (2025)	Capability	Review of LLMs as research subjects	LLMs as cognitive models face validity and generalisation challenges	Qualitative assessment	N/A; review
Organisciak et al. (2023)	Assessment	AUT scoring; LLM vs. human raters	LLM scoring greatly improves over semantic distance methods	ICC = 0.86 (vs. human raters)	High; ICC validated
Beaty et al. (2022)	Assessment	AUT; semantic distance recommendations	Guidelines for reliable automated originality scoring	r ≥ 0.70 (validity)	High
Saretzki & Benedek (2026)	Assessment	DT scoring; validity evaluation	Automated scoring shows mixed validity evidence across DT indices	r = 0.48–0.82 (validity range)	Medium; mixed validity
Goecke et al. (2024)	Assessment	Scientific creativity scoring (German)	LLM-based scoring cross-validated with human ratings	ICC = 0.81	High; ICC validated
Yang et al. (2023)	Assessment	Chinese DT; TransDis model	Transformer-based model for Chinese	r = 0.75 (vs. human)	High; cross-cultural validation

Demszky et al. (2023)	Assessment	Review of LLMs in psychology	DT assessment LLMs as scalable tools for psychological text analysis	Qualitative framework	N/A; review
Lin (2026)	Assessment	Validity-guided workflow	Framework for robust LLM research in psychology	Methodological guidelines	N/A; methodological
Rezwana & Maher (2023)	Co-Creativity	COFI framework; human–AI interaction	Framework for modelling co-creative interaction	Theoretical framework	N/A; theoretical
Wang et al. (2026)	Co-Creativity	Combinational generative model; co-ideation	Human–AI co-ideation enhances idea novelty	d = 0.56 (novelty gain)	Medium; small sample
Magni et al. (2024)	Co-Creativity	Creativity evaluation; bias study	Humans biased against AI creativity (creativity gatekeeping)	d = 0.47 (bias effect)	Medium; between-subjects
Lazovsky et al. (2025)	Co-Creativity	Question asking; prompt engineering	Creative inquiry links question asking to prompt engineering	Theoretical framework	N/A; theoretical
Park & Choo (2024)	Co-Creativity	Prompt engineering; education	Practical strategies for effective prompt design	Qualitative guidelines	N/A; educational
McCoy et al. (2023)	Methodology	RAVEN framework; novelty evaluation	LLMs copy from training data; novelty varies by task	Copy rate = 12–24%	High
Kostikova et al. (2026)	Methodology	Survey of LLM limitations	Data contamination, hallucination, and bias remain key challenges	Qualitative survey	N/A; survey
Wingström et al. (2024)	Ethics	Perspective survey; CS vs. artists	Divergent views on redefining creativity in the AI era	Qualitative survey	N/A; survey

Runco & Jaeger (2012)	Foundation al	Theoretical review	Standard definition of creativity: novelty + usefulness	Theoretical framework	N/A; foundational
Kozbelt et al. (2010)	Foundation al	Theories of creativity review	Comprehensi ve taxonomy of creativity theories	Theoretical framework	N/A; foundational
Page et al. (2021)	Foundation al	PRISMA 2020 guideline	Updated reporting standard for systematic reviews	Reporting guideline	N/A; guideline

To address the methodological concern that systematic reviews require transparent quality assessment, we evaluated all included empirical studies against a set of adapted criteria (Table S3). These criteria were derived from the PRISMA 2020 risk-of-bias framework and the experimental design standards for psychological research, modified for the specific context of LLM-creativity studies.

Table S3. Quality assessment criteria for included empirical studies in LLM-creativity research.

Criterion	Description	Application in This Review
Experimental Design	Random assignment, control conditions, pre-registration of hypotheses and analysis plan	Studies with pre-registered protocols or random assignment were rated 'High'; studies with quasi-experimental designs or post-hoc comparisons were rated 'Medium'
Sample Size	Adequate statistical power (N ≥ 100 for between-subjects comparisons, or N ≥ 50 per group); reporting of power analysis	Studies with N ≥ 100 or reported power analysis were rated 'High'; studies with N < 50 or no power analysis were rated 'Medium' or 'Low'
LLM Specification	Clear reporting of model version (e.g., GPT-4, GPT-3.5), temperature, max_tokens, and system prompt	Studies with full LLM specification in main text or supplementary materials were rated 'High'; studies with incomplete reporting were rated 'Medium'
Blinded Scoring	Human raters blinded to the source (AI vs. human) of responses; use of automated scoring with validated algorithms	Studies with blinded human raters or validated automated scoring were rated 'High'; studies with unblinded scoring or unvalidated algorithms were rated 'Medium'
Fluency Matching	LLM output quantity matched to human output quantity (e.g., 10 responses per participant) to avoid	Studies with fluency-matched comparisons were rated 'High'; studies comparing unconstrained LLM output to human output were rated

	confounding fluency with originality	'Medium' (noted as a limitation)
Control Variables	Controlling for participant demographics, prior AI experience, and task order effects	Studies with reported control variables were rated 'High'; studies with no control variables were rated 'Medium'
Replicability	Availability of materials (prompts, code, data) in supplementary materials or public repositories	Studies with open materials or detailed supplementary information were rated 'High'; studies with limited methodological detail were rated 'Medium'
Publication Status	Peer-reviewed journal article vs. preprint or conference proceedings	Peer-reviewed articles were rated 'High'; preprints were rated 'Medium' (noted as provisional)
Training-Data Contamination Risk	Whether the evaluated stimuli could have appeared in the model's training data, graded by corpus transparency (documented vs. undisclosed) and stimulus provenance (post-cutoff, dynamically generated, or classic)	Studies using post-cutoff or dynamically generated stimuli with documented corpora were rated 'High' (low risk); studies using classic tasks (e.g., AUT, TTCT) with proprietary models were annotated as carrying high exposure risk and their conclusions qualified accordingly (noted as a limitation)

Note. The quality assessment was conducted independently by two authors. Disagreements were resolved through discussion. Studies rated 'High' on all applicable criteria were classified as providing strong evidence; studies rated 'Medium' on one or more criteria were classified as providing moderate evidence with noted limitations. No study in the current literature meets all 'High' criteria, reflecting the nascent state of the field.

Table S4. Row-level effect-size derivations for Table 2.

Row	Study and measure	Source statistic	n (LLM vs. human)	Conversion	d	g	95% CI
1	Hubert et al. (2024), AUT originality	Open raw data (OSF: osf.io/xv6kh), participant-level means after reconstructing the authors' fluency matching: human M = 0.737 (SD 0.040); GPT-4	151 vs. 151	g = J(M2−M1)/Spooled	2.61	2.61	[2.30, 2.91]

		M = 0.816 (SD 0.014)					
2	Hubert et al. (2024), AUT elaboration	Open raw data: human M = 3.36 (2.61) words per response; GPT-4 M = 12.87 (2.01)	151 vs. 151	as row 1	4.08	4.07	[3.68, 4.46]
3	Hubert et al. (2024), Consequences originality	Open raw data: human M = 0.667 (0.048); GPT-4 M = 0.718 (0.012); the published Consequences ANOVA duplicates the AUT values (apparent reporting error), so raw data were used	151 vs. 151	as row 1	1.47	1.47	[1.22, 1.72]
4	Grassini & Koivisto (2025), FIQ flexibility	Published Table 1 session-level descriptives: human M = 0.899 (0.063); AI M = 0.934 (0.023)	246 vs. 247	as row 1	0.74	0.74	[0.56, 0.92]
5	Grassini & Koivisto (2025), FIQ subjective creativity	Published Table 1: human M = 0.738 (0.083); AI M = 0.632 (0.080)	246 vs. 247	as row 1	−1.31	−1.31	[−1.50, −1.11]
6	Koivisto & Grassini (2023), AUT originality	Published Table 1 mean semantic distance: human M = 0.91 (0.06); AI M = 0.95 (0.03); sensitivity: mixed-model B = 0.049,	256 vs. 33	as row 1	0.70	0.70	[0.33, 1.06]

		**t(274) = 4.95 → d ≈ 0.60**					
**7**	**Bellemare-Pepin et al. (2026), DAT vs. population average**	**Open data: GPT-4 (default temperature) valid responses M = 83.99 (3.03); random subsample of 500 from N = 100,000 human scores, M = 81.16 (7.14)**	**503 vs. 500**	**as row 1**	**0.52**	**0.52**	**[0.39, 0.64]**
**8**	**Bellemare-Pepin et al. (2026), DAT vs. top 50%**	**Open data: random subsample of 500 from the top 50% of human scores, M = 86.80 (3.34)**	**503 vs. 500**	**as row 1**	**−0.88**	**−0.88**	**[−1.01, −0.75]**

Note. Spooled = pooled standard deviation with unequal-n weighting; J = 1 − 3/(4(n1+n2)−9) (Hedges' correction); CI = g ± 1.96 × SE with SE = √((n1+n2)/(n1n2) + g²/(2(n1+n2−2))). Derivations 1–3 used the primary study's open raw data with the authors' fluency matching reconstructed. Derivation 6 replaces the earlier "flexibility" row (the primary study did not measure flexibility) and serves as the restored fluency-matched comparison (the AI was prospectively constrained to human response counts). Derivations 7–8 replace the earlier non-derivable top-quartile row; human subsamples were drawn with a fixed seed (20260822). Analysis code is available from the corresponding author on reasonable request.

GCA = Generation-Capability-Assessment; AUT = Alternative Uses Task; TTCT = Torrance Tests of Creative Thinking; DT = Divergent Thinking; ICC = Intraclass Correlation Coefficient; d = Cohen's d; r = Pearson correlation; FC = Functional Connectivity. Effect sizes are reported as in the original studies. Quality ratings are based on the criteria described in Table S3: High = pre-registered design with adequate sample size, blinding, and control variables; Medium = adequate design but with some methodological limitations (e.g., no fluency matching, small sample, unblinded scoring); N/A = not applicable (theoretical, review, or methodological papers).
